# Too Early to Tell: The Potential Impact and Challenges—Ethical and Otherwise—Inherent in the Mainstreaming of Dharma in an Increasingly Dystopian World

**DOI:** 10.1007/s12671-017-0758-2

**Published:** 2017-06-29

**Authors:** Jon Kabat-Zinn

**Affiliations:** Center for Mindfulness, University of Massachusetts Medical School, 55 Lake Avenue North, Worcester, MA 01655 USA

## Introduction

It is said that Zhou Enlai, the Chinese Premier, who as a young man was a major force in the Chinese Revolution, when asked late in life by a journalist for his thoughts about the legacy of the French Revolution, replied “Too early to tell.”^1,^ ^2,^ ^3^

It became a meme of sorts, even if it was based on a misunderstanding. I love the whole notion that it may be too early to tell—about a lot of emergences in our world. But sometimes, we need to act forcefully and with resolve, based on the best projections for what may take place given various lines of broadly accepted scientific evidence (such as the global receding of the glaciers and the melting of the polar ice caps) and its modeling algorithms, even if we cannot be sure of just how bad bad could be, such as in the case of global warming. By the time it plays out in real time, any action is already too late. My late Korean Zen teacher, Seung Sahn Seon Sa, was fond of saying, meaning just that, “The arrow is already downtown.” Whether the overwhelming evidence for global warming is denied by politicians in any given moment out of cynicism, ignorance, or greed is quite another story.

So perhaps at this moment in time, it is way too early to tell what the likely fate of humankind will be, given our self-destructive, aggressive, violent, tribal, dualistic, and delusional tendencies as a species, in spite of all the civilizations, diverse cultural flowerings, beauty, understanding, wisdom, and compassion and basic human goodness that humanity has also brought to the planet in the very short arc of human history—say, to be generous, perhaps 400 generations since the last ice age. British historian, Arnold Toynbee, famously said that in the future, the coming of Buddhism to the West would be seen as the signature historical event of the twentieth century. Maybe it is even now, half a century later, way too early to tell.

A major koan in the Chan tradition, over fifteen hundred years old: “What is the meaning of Bodhidharma’s coming from the West?”^4^ One credible answer: “Too early to tell!” Even now.

I would say that the same is true of the mainstreaming of mindfulness in the world as both a practice and a way of being. In terms of the work of MBSR (mindfulness-based stress reduction), to say it right off the bat, since it is increasingly questioned by people unfamiliar with it in practice, the mainstreaming of mindfulness in the world has always been anchored in the ethical framework that lies at the very heart of the original teachings of the Buddha.^5^ *Sila*, meaning “virtue” or “moral conduct” in the Pali language, is represented by the third, fourth, and fifth factors of the Eightfold Path (the fourth of the Four Noble Truths): wise/right speech, wise/right action, and wise/right livelihood. While MBSR does not, nor should it, explicitly address these classical foundations in a clinical context with patients, the Four Noble Truths have always been the soil in which the cultivation of mindfulness via MBSR and other mindfulness-based programs (MBPs) is rooted, and out of which, it grows through ongoing practice. More on this to follow, in terms of the both Hippocratic Oath and the Bodhisattva Vow. Parenthetically, MBSR was also designed from the beginning as a vehicle of right livelihood for the people who would be drawn to become MBSR instructors.^6^

Now that mindfulness already has a multidecade track record in the mainstream of medicine, health care, as well as increasingly in other societal avenues, however, nascent at this particular time, from the law to education to government, to criminal justice, to sports, perhaps the major challenge for all of us who practice the dharma in one form or another and care deeply about suffering and the end of suffering and the root causes of suffering is to contribute optimally and skillfully to the ongoing development, refinement, articulation, and embodied authenticity of dharma wisdom in all the various domains within which it is taking root, whether within the mainstream or within more Buddhist-oriented streams. After all, the mainstreaming of dharma through mindfulness is *prima facie* a positive and healing occurrence and a tremendous opportunity for addressing some of the most fundamental sources of pain and suffering in our world at this moment in time. That would include the Orwellian distortions of truth we are now seeing on a daily basis in the news, and the perpetuation of dystopian “governance” by seemingly elevating greed, hatred, and delusion to new heights, with all its attendant consequences for the fragility of democratic institutions.

**Fig. 1:**
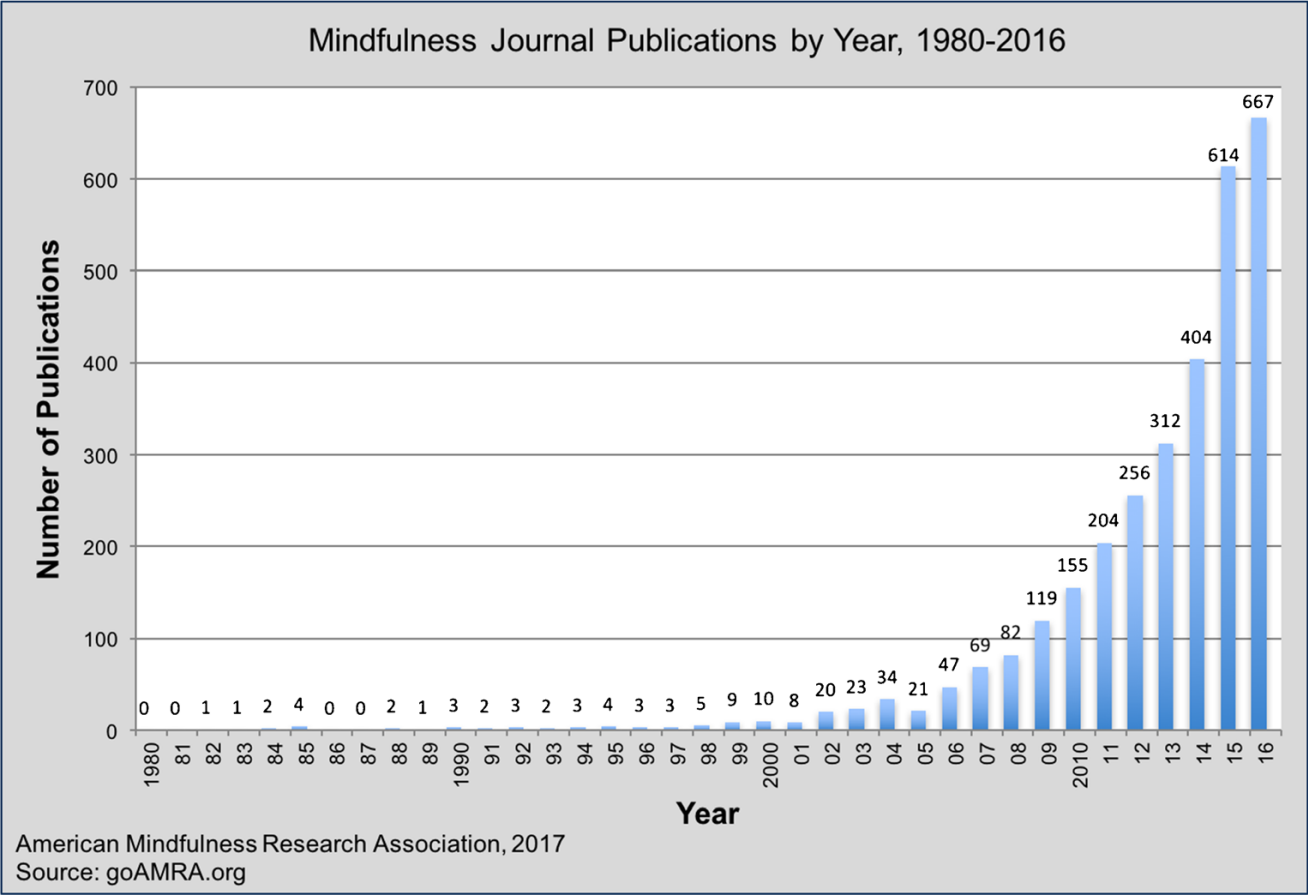
Number of publications on mindfulness by year

My guess and profound hope is that as planetary citizens and devoted mindfulness practitioners, we all have necessary, even critical roles to play in what unfolds from here, however, small or insignificant we might think our contribution might be when we begin, especially in light of the mega-geopolitical forces playing out in our time through terrorism, war, cynicism, and death around the world, and the historical roots that feed such forces within the human family and how easily these are invoked and played upon by demagogues. The evidence of the flowering of mindfulness in this era (see Fig. 1 as one indicator) is widespread. It can be gleaned from the depth and breadth of research articles in the journal *Mindfulness* and increasingly throughout the top tier scientific and medical literature, in the documenting and curating of this exponential flowering in the Mindfulness Research Monthly, and in the various series of edited volumes in *Mindfulness and Behavioral Health* being put out by Springer, including the latest volume, devoted to Mindfulness and Ethics (Purser et al. 2018). The very fact that a major scientific publisher thinks the subject of mindfulness and ethics is relevant enough to invest its resources to bring this topic into this conventional form of mainstream academic discourse is significant, as is the fact that there are so many different credible voices and perspectives being expressed from vastly different backgrounds. Nirbhay Singh is to be congratulated on his leadership in generating these vehicles for reporting ongoing research and offering a range of opinion pieces and perspectives on the field, and even more for helping to develop the field itself and the ongoing discourse and inquiry that are evolving through his launching of the journal *Mindfulness*, and of the *Mindfulness in Behavioral Health Series* through Springer. Academic volumes may not change the world all that much, but they sometimes put their finger on the pulse of emergent possibilities in science and medicine that can augur transformative changes in planetary culture.

What I intend to do in this paper is offer a non-exhaustive perspective on the original core aspirations, as I experienced them, behind introducing mindfulness as a practice and as a way of being into the mainstream world, initially through medicine and health care in the form of MBSR, and then—as the meme and practice took root and interest spread with increasing evidence of its multifaceted efficacy—to varying degrees into education, business, social justice, politics, and the more global domain of acknowledging a moral and exceedingly practical responsibility for the planet on the part of our species as a whole. For me, this trajectory has always been one of generating an ever growing number of hopefully skillful approaches for effectively addressing widespread suffering and its root causes in the human mind. These classically take the form of (1) greed; (2) fear and aversion, and the distain, enmity, and vilifying that frequently accompany them, including the racial/ethnic dehumanizing phenomenon of “othering”; (3) delusion, namely, mistaking appearance for reality; and (4) the toxicity, ignorance, and blindness that arise from ignoring intrinsic human values such as kindness and compassion, and the humanity in others. When these intrinsic human values are ignored, their absence tends to undermine our speech, our actions, our choices and sources of livelihood, and thus, potentially, our ethical stance and moral core as human beings. That absence also undermines our social fabric as democratic communities and our commitment to value, if not celebrate, both commonality and difference in pluralistic societies.

That said, an unintended but in retrospect inevitable consequence of the speed and effectiveness with which mindfulness has moved into the mainstream of so many disciplines and aspects of life in the past decade is that the very word “mindfulness” is now at some risk of losing its meaning, as a small minority of people, mostly out of ignorance or opportunism, I am guessing, apply it to advertise and sell dubious products that have little or nothing to do with mindfulness and, in some cases, promote seemingly exploitative agendas in business and elsewhere. In all likelihood, they have no idea that mindfulness is rooted in an ancient and arduous meditation practice and an ethical soil. My hope is that this is a temporary and self-limiting phenomenon that will remedy itself as the ongoing dynamic of mindfulness/universal dharma moves ever more deeply into the mainstream of society and contributes to shaping the minds and understandings and communities of those who are deeply touched by the practice itself. Our ongoing debates and dialogs on the subject in the growing global mindfulness community can contribute to that larger aim.

## Working Definitions of Mindfulness:

Just so, it is clear what I mean when I use the word “mindfulness,” I am using it as a synonym for “awareness” or “pure awareness.” The operational definition that I offered around the work of MBSR and the intentional cultivation of mindfulness (or access to mindfulness) is that mindfulness is *the awareness that arises from paying attention, on purpose, in the present moment, and non-judgmentally* ^7^. Non-judgmentally does not mean that there will not be plenty of judging and evaluating going on—of course there will be. Non-judgmental means to be aware of how judgmental the mind can be, and as best we can, not getting caught in it or recognizing when we are and not compounding our suffering by judging the judging. Of course, awareness is an innate, constitutive, and defining aspect of our humanity. It is at least as powerful and useful as the faculty that allows for thought and emotion, because it affords a momentary opportunity to hold those energies from an additional vantage point or dimension of experience (a knowing that includes but also transcends the merely conceptual), thus, promoting the possibility of discernment and wisdom. The practice of mindfulness includes coming to recognize the faculty of awareness within oneself and learning how to befriend and inhabit it as the primary dimension/abode of experience, a faculty that can be an effective and liberating counterbalance to the also very powerful and creative, although too often imprisoning and blinding faculty of thinking (and the emotions that accompany our thoughts, likes and dislikes, memories, and anticipations). These two modes both benefit from collaborating seamlessly together. Moreover, awareness has the potential, with a greater ease of recognition and access, to become the default mode of our moment-to-moment experience, rather than its opposite, mindlessness, or mind wandering (Killingsworth and Gilbert 2010). It should also be noted that, since in Asian languages, the word for “mind” and the word for “heart” are usually the same, we cannot fully understand the word “mindfulness” in English without simultaneously hearing or feeling the word “heartfulness.” They are one and the same. Thus, the meditative cultivation of mindfulness, whether formally or informally, involves intentionally, as best we can, bringing an openhearted and affectionate attention to our experience. This points to the essential non-separation between mindfulness and compassion, at least the way I understand it within the framework of MBSR.^8^

Another way to frame “mindfulness” that I have found useful is that it subtends the multidimensional domain of relationality itself. We can bring awareness to the question of who is speaking when we say things like “I have a body” or “I am breathing” or “I am thinking” or “I am meditating.” The non-trivial question of “Who am I?” points to wakefulness itself, and non-separation, to the mystery of lived experience and sentience, and the artificial separation inherent in subject/object duality.

Taking the non-dual perspective into account suggests that it is important to thread the intrinsic complementarity of the instrumental and non-instrumental dimensions of mindfulness together from the beginning both in one’s own practice and in one’s teaching, if one is in a position to offer instruction in the practice of mindfulness.^9^ The instrumental dimension puts learning to meditate in the same domain as any other learning, such as to drive a car, or play a musical instrument—you learn the method, work at it diligently, make progress toward targeted goals, and eventually develop some degree of familiarity or even mastery. The non-instrumental reminds us that with meditation, from the very beginning there is also, seemingly paradoxically, no place to go, nothing to do, and nothing to attain—no special state, insight, or way of being. This is because every experience is already special if held in awareness. Being alive is special. Experiencing anything is special if it is recognized as pure experience without fabricating an “experiencer” and a subsequent self-centered and perforce limited narrative to support it. Neither the instrumental nor the non-instrumental domain by itself is sufficient to embody wakefulness, wisdom, and compassion or to help others to uncover these dimensions of being in themselves. Together, like two strands of a double helix, they support each other.^10^

## Are Mindfulness-Based Interventions Watered-Down Dharma?

In 1991, I was asked the following question by a journalist working for a Buddhist magazine (Tricycle, in its first year):

What are the implications of taking mindfulness practice outside of its formal traditions and historical context? Is there a danger of watering it down too much, of endangering its integrity?

This is how I responded in 1991:

I think there is always a danger of a tradition losing its integrity or being distorted by someone whose understanding is limited. But it’s even more of a danger, if one is concerned about human suffering, to become so doctrinaire that you’re basically captivated by your own requirements to be pure to a particular tradition. Religious traditions are famous for their parochialism. Lots of Buddhists get into ego trips, such as “My practice is better, deeper, faster, more complete, or more spiritual than your practice.”

In 2016, I was invited to revisit the same question for the magazine’s 25th anniversary edition. Their invitation stated:

The idea would be that we ask you this question again, today, after so much has happened with the mindfulness movement. What are your thoughts on the way this is playing out? Has anything surprised you? You have been an important figure in this development, but now it’s gotten so big. What do you make of the whole thing? Is there a next phase of development that you would like to see?

What follows is my response to that same question, 25 years later, modified and expanded upon for the purposes of this paper and the collective inquiry and dialog taking place at this point in time throughout the mindfulness community.

This is a big question, and rightly so given the increasing buzz and hype around mindfulness in the commercial and on-line worlds at this moment. To address it even partially, we will also need to take into account and honor the seriousness, the depth, and the authenticity with which mindfulness has taken root in mainstream culture in so many different domains in so many different parts of the world. So my response will be quite a bit longer than in 1991 (which was 2 years prior to the airing of the Bill Moyers PBS Special, *Healing and the Mind*, seen by over 40 million viewers, in which MBSR was featured in one 45-min segment), a time in which very few people in the mainstream culture understood that the word “mindfulness,” if they knew it at all, had anything to do with an ancient meditative discipline and tradition, or that it might be infinitely deep and have profound life-transforming and even world-transforming implications. It was also a time in which anything having to do with meditation or yoga was considered the far side of the drug-crazed lunatic fringe by mainstream culture, so there were major obstacles to any attempts to make the practice of either understandable and commonsensical within society as a whole.

We can see that the original wording of the question Tricycle posed in 1991 carried an implicit assumption, an assertion that the dharma was of necessity being “watered down” to one degree or another in MBSR, and the question was framed as: “When is it “too much?” It also included concern for mindfulness being taken out of its historical, cultural, and religious contexts, thereby, possibly endangering its integrity and depth. These are increasingly important questions in this era and certainly merit examining and debating, as I believe our community is doing more and more. They are also deep, multidimensional questions, not amenable to brief and facile responses.

To get started, what if we posited for a moment that, in essence, the dharma (the lawfulness that the Buddha discovered, described, and offered skillful methods for developing [*bhavana*]) is not being watered down (whatever that might mean—Let us not forget the Zen Master who described his efforts as “selling water by the river,” and “totally without merit”) (Kapleau 1965) and that whatever we mean by “historical, cultural, or religious context” is era-dependent? What if in this era, mindfulness has been contextualized adequately and appropriately—or adequately and appropriately enough—in the domains within which mindfulness training of one kind or another, some of it nascent, is taking place in mainstream settings—medicine and health care, K-12 schools, college and professional schools, corporations, the law, prisons, the military, policing, social agencies, and government—at least up to now? Revisiting the question might be especially timely, given that the boundaries between the mainstream and the Buddhist world are so much thinner and less distinct than they were 25 years ago—witness Tricycle’s reach, and that of other Buddhist publications, the Dalai Lama’s global prominence, the influence of the Mind and Life Institute over the past 30 years, *Mindful* magazine, this journal, and so many other currents in the mainstream world. What is more, the urgency and the need are, if anything, more pressing than ever—witness the Black Lives Matter movement in the USA as a response to endemic racism and police violence in poor neighborhoods, the fear and mistreatment of immigrants, particularly the so-called “undocumented,” the underlying and tenaciously persistent social and economic injustices in our inner cities, the energy and pipeline wars pitting the power of the state against indigenous people on their own land, the growing concerns about the accessibly of clean water, the ascendency of Trump and the forces and values he represents, and the growing mindset of populism around the globe that is grounded in many deep and legitimate grievances, but that is also readily exploited and potentially betrayed by cynical manipulation.

This may indeed be a pivotal moment for our species to come to our senses both literally and metaphorically in terms of mobilizing and operationalizing in the mainstream world and its institutions what we know to be the intrinsically healing, illuminating, and potentially liberating virtues and power of mindfulness, both as a practice and as a way of being. In embarking on such a path, we might transcend or at least learn how to work more imaginatively, creatively, and with good will with the tyranny of our own thoughts in the form of conventional dualisms, such as right/left, liberal/conservative Democrat/Republican, rich/poor, true/false, the good guys and the bad guys, and even sacred/secular, which may all be, in their own domains, true to a degree, but not true enough to bring about either healing or peace or compassion or wisdom at the levels that the planet, our species and many many others, are calling out for. More on this below.

Perhaps the question “What is called for now?” in 2017, in terms of wise and compassionate action for each of us individually and all of us collectively as contributors to and participants in this broadening conversation in both scholarly and practitioner (in all senses of the word) circles might be a worthy koan at this moment, where, for whatever reasons, there is a risk of falling into reflexive parochialisms. A collective inquiry in the spirit of good will, deep listening, and an appreciation for the inclusivity of the dharma is especially important if we care about nurturing a wiser and more compassionate world at a time when discovering and drawing upon our deepest innate resources as human beings (our “true nature” we might say) may, without exaggeration, be critical to the survival of our world and of our species and many others.

We know that there are as many doors into wakefulness and embodied wisdom as there are human beings. Even within the framework of Buddhadharma, everybody’s unique trajectory starts of necessity with encountering it within one culture, or tradition, or school or person—one of a host of other pathways and perspectives that may also play their roles at some point in a person’s life in eventually coming to understand the teachings to some degree, always in flux, embody them as best one can, and live them wholeheartedly through ongoing learning, growing, opening, and ultimately, coming to terms with things as they are, which has nothing to do, just to be clear about it, with passive resignation, but rather with discernment and enaction^11^ in whatever ways are deemed appropriate to the circumstances, always changing of course. To be explicit, I mean that the underlying motive force for this work is the intuition, the longing, and the very real possibility of liberation from greed, aversion, and delusion on the individual, institutional, and global level, nothing less. This first requires that we recognize those factors in our own lives and minds when they arise and learn through practice how to not be so caught by them. Politically, that might mean developing a democracy 2.0 based on the Hippocratic principle to first do no harm, and grounded in the lawfulness that a universal dharma foundation based on widespread embodied practice might provide. If democracy is truly based on the rule of law, and the law itself were grounded on the first principle of minimizing harm and maximizing wellbeing for all members of the body politic, writ large and understood broadly—a kind of political, social, and economic Hippocratic Oath—then the lawfulness of the dharma might well provide an inescapable and essential foundation for upgrading the wisdom inherent in our laws and institutions at present to be adequate to the age in which we find ourselves—if we are to face and deal mindfully and heartfully with the ways in which we can be driven primarily by fear and ignorance rather than by love and wisdom. A recognition of the process of “selfing” and learning how not being so caught in its gravitational pull is essential for us to find a new way to transcend those mind states that distort experience and further separation as opposed to inclusion and a larger “at-home-ness” that allows for discomfort without it leading to scapegoating and violence, but rather more in the direction of kindness and mutual flourishing.

This being so, we might ask whether we can usefully and in all humility differentiate Buddhadharma from a more universal articulation of that very same dharma that might serve as a door into insight and potential liberation from stress and suffering of all kinds for those for whom the Buddhist doors are not going to be readily accessible? That has been our intention in MBSR from the very beginning, so I stand by my response of 25 years ago. Non-dual Mahayana teachings suggest that a direct transmission outside the sutras is possible,^12^ and that all of the dharma is, we might say, holographically embedded in any one element of the dharma.^13^ In the face of suffering, how much exposure to mindfulness (also understood as *heartfulness*) would be too little, or too “decontextualized,”^14^ if it inspired or propelled somebody who was suffering in one fashion or another to practice mindfulness both formally and informally and find new ways to be in a wiser relationship to the actuality of his or her situation, inwardly and outwardly, until there is no absolute separation between inner and outer?

I recognize that critics can object that “mindfulness” as we use the term may not be “right mindfulness” (*sammasati*)*.* This is a topic for further conversation, beyond the scope of this paper. Let me just assert that we consider what we teach in MBSR and other mindfulness-based interventions to be “wise” or “right” mindfulness, to whatever degree we can manage to embody and convey it, and keep it in the forefront of our awareness.^15^ MBSR was meant to be a potentially skillful and potent glide path into the heart and essence of dharma wisdom, a first exposure at least, and a direct first-person one at that, based entirely on practice and empirical investigation of one’s direct experience. But it was not, nor was it ever meant to be a vehicle for teaching Buddhism per se, disguised, “stealth,” or of any other variety. Still, it bears keeping in mind that Buddhism itself was and continues to be an evolutionary and historical development, and that the Buddha himself was, arguably, not a Buddhist.

## MBSR As One of an Infinite Number of Possible Skillful Means

The whole idea from the beginning of MBSR, now in its 38th year (by one count, there are over 740 programs in hospitals and medical centers worldwide^16^) was to make mindfulness meditation practice so commonsensical to people facing stress, pain, and chronic illness that they would actually incorporate it into their lives *as a practice and a way of being* on a more or less daily basis long enough to see for themselves whether it was of some value in facing and befriending suffering—and in the process, understanding the nature of their own suffering to one degree or another over time and perhaps even freeing themselves from its root causes.^17^ MBSR (and hopefully the same can be said for its many mindfulness-based cousins^18^) was always meant to be a skillful means for making the universal essence of dharma, or at least a first taste of it, accessible to virtually anybody who cared to explore it, thereby, hopefully reducing the barriers to ongoing wakefulness, embodied kindness, and wisdom in human beings, whatever their views, convictions, and personal history.^19^

From the start, originating within a hospital and academic medical center, MBSR was of necessity rooted in the ethical soil of the Hippocratic Oath, namely, to first do no harm. This truly noble vow that each doctor takes upon graduating from medical school, has undergirded medicine and health care, at least in principle, from their very beginnings. It was further elaborated by Maimonides in the twelfth century in Egypt. Succinctly put, the patient’s needs come first, before the needs of the doctor and other caregivers. There is a foundation of selfless action built into this medical ethos, akin to the Bodhisattva Vow, namely, to work for the liberation of others with all one’s energy, putting their liberation above one’s own. Still, returning to *primum non nocere*, it is important to ask how, practically speaking, would one even know if one was doing harm in one way or another, either by commission or omission, without a high degree of mindfulness on the part of the physician, especially during the doctor-patient encounter?^20^ The same might be said for assessing what the patient’s needs might truly be. We can see that mindfulness inevitably lies at the heart of the Hippocratic principle, as does heartfulness/compassion. And it is an *oath*, so in that sense, a sacred vow if we take it at face value, which means seriously and personally. And it includes one’s own wholeness as a caregiver, so from the non-dual perspective, kindness and clarity extended to the other is inseparable from kindness and clarity extended to oneself.

## Heterogeneity Among MBSR Teachers and Participants

Regarding the depth and authenticity of the dharma roots of MBSR and their actual embodiment in practice, as with any educational or clinical program or curriculum, there is inevitably a heterogeneity in the quality and depth of its delivery and in the container within which it is unfolded, especially since none of us, as far as I know, claim any special attainment. The quality of any MBSR program depends critically on the embodied presence, understanding, and lived experience of the instructor including, of course, the rigor and depths of her or his (1) own training trajectory with dharma teachers, (2) personal meditation practice, (3) understanding of the dharma, and (4) motivation to do this kind of work in the first place. Even among well-trained and highly motivated MBSR instructors, as with any other field, there will always be a spectrum of competencies, strengths, and weaknesses each of us will encounter as we work to continually develop ourselves as authentic teachers and as human beings, hopefully receiving ongoing support from the greater sangha of fellow MBSR instructors and our other teachers. In parallel fashion, a group participating in an MBSR program together will always show a range of benefits, from nothing or even getting more encumbered by one’s problems and challenges, to new and possibly liberating ways of being in relationship to the present moment, silence, awareness, stress, pain, and social situations. Not every person taking an MBSR program will go on to cultivate a deeper dharma practice, although many do. And yet the “seed” of mindfulness as a way of being can invisibly take root in one’s heart by the end of 8 weeks of participation in a MBSR program, or even before, and may be carried there in some fashion, even in the absence of a regular practice or a conscious remembering to bring it into everyday life circumstances.

Moreover, as with any educational curriculum, not every teacher is necessarily capable of inspiring a lifetime of ongoing practice, inquiry, and deepening development in others. Quantitating such variables and their consequences both retrospectively and prospectively would certainly be a valuable contribution to future research. At this juncture in time, the global MBSR and mindfulness-based cognitive therapy (MBCT) teacher communities are taking great pains to establish minimal teacher competency standards and criteria as well as high-level professional education to maximize their long-term impact on the wellbeing and continued development of the lives of participants in 8-week-long mindfulness-based programs. In a number of professional training programs, Buddhist meditation teachers with interest in contributing to high dharma integrity in mainstream mindfulness programs, such as MBCT, are an integral part of these efforts.^21^

## Possible Long-Term Effects Within Society

From its inception, MBSR was conceived as a public health intervention rather than a therapy, designed to, over time, move the bell curve of society as a whole in the direction of greater health, wellbeing, and wisdom through tapping the deep interior resources and reservoirs of the heart and mind and body native to all of us, by virtue of being human, for learning, growing, healing, and transformation, as well as the social connectedness and learning from each other that spontaneously arise in class-like settings, when room is made for such, to catalyze and promote health in every dimension of that term. Reflecting back over the past 38 years since the inception of MBSR, there are many thousands of people who now have an ongoing meditation practice because of having taken a mindfulness-based program at some point in the past. One signature characteristic of undertaking an MBSR program is that, by design, it requires an immediate lifestyle change in the form of devoting significant time each day to formal mindfulness meditation practices (the body scan, sitting meditation, and mindful hatha yoga) as well as a range of informal mindfulness meditation practices throughout the day in virtually any and all life situations.

In this way, MBSR serves as a catalyst for exposing large numbers of people in society to meditation practice, as well as a portal into a lifetime of ongoing practice for many. Beyond that, there are now many hundreds if not thousands of clinicians, researchers, graduate students, young scientists and physicians, and other professionals who are committed to the cultivation of mindfulness in their own lives, often within various Buddhist traditions. Some are also ardent students of its root texts, teachers, and teachings. But virtually, all of them are working in mainstream (I avoid the term “secular” as best I can, as in “secular mindfulness,” since it feels dualistic to me, and surrenders the sacred^22^ element of practice and embodied wakefulness, something I am not willing to do) settings to bring a greater mindfulness and heartfulness into the culture in a broad range of different ways and in many different domains. As noted above, this includes medicine, health care, public health, primary, secondary, and “higher” education, business, the legal profession, the tech world, professional and amateur sports, social activism and social justice, criminal justice, the military, and government, to name a few. Some are nascent efforts, while many have been in place for years and are thriving. For example, the Parliament in the UK has a popular 8-week mindfulness program for members of the House of Commons and the House of Lords (at the time of writing, over 140 parliamentarians have already taken the program). It recently issued an All Party Parliamentary Report^23^ recommending major mindfulness initiatives in four areas of national interest: health care, education, business/innovation, and criminal justice. Nothing like this has ever happened before. They are now in the process of reaching out to Parliamentarians in other countries to develop a Global Mindfulness Initiative.^24^

In the USA, Congressman Tim Ryan (D, Ohio) wrote a book entitled *A Mindful Nation* that inspired the title of the UK report (Ryan 2012). He is still in office. This nascent trend within government is now of even greater import in the face of Brexit, the Trump presidency, and other nations’ moves in a similar direction.

In a telling parallel unfolding that underscores the interweaving strands of various traditions within the more universal framework and underpinnings of mindfulness-based interventions, some prominent MBSR teachers and researchers were formerly or remain decades-long students of senior Buddhist teachers in various traditions. Others completed a traditional 3-year retreat, or in a few cases, multiple 3-year retreats, yet were motivated following that experience to train to become MBSR teachers. And some MBSR teachers or teachers-in-training are currently senior Chan monks and nuns from China and Taiwan. How are we to understand this phenomenon and the desire among Buddhist meditation teachers to teach MBSR, especially if MBSR suffers from the various flaws that some suggest that it does, including being unethical, or engaging in inappropriate and potentially harmful “cultural appropriation?”

To my mind, these and many other emergences suggest manifold and virtually limitless opportunities for further cultivation and development by people who have a deep love for the dharma and a deep understanding of it through both scholarship and personal practice. The need is infinite, so why not help build an ever-self-correcting edifice of ongoing learning, wisdom, and compassion in the world rather than assume, usually wrongly, that mainstream efforts such as mindfulness-based programs—if they are indeed what we mean by calling them “mindfulness-based” in the first place—are fundamentally unethical, dumbed-down, or divorced from a deep liberative potential? Even if there are specific instances where problems need to be addressed, as of course there always are, does that mean we should abandon the essence? Hardly. Problems, no matter how challenging, might be seen as rich opportunities for ongoing learning on all our parts within a robust ethical framework and ongoing collective inquiry. Iterative interaction and cooperativity, this is how things evolve on this planet, and within the brain and social systems…there is no one right way. There are always a multiplicity of interacting, self-illuminating streams, much like ongoing neural-net algorithms that evolve over time from massive iterative inputs and outputs.

## The Stress of Success

Figure 1 shows the growth in the number of papers per year since 1980 in the medical and scientific literature that have the term “mindfulness” in the title.^25^ It is immediately apparent that mindfulness has become a “field” of research and clinical practice with a body of increasingly high quality scientific evidence in support of its various potential applications and effects. This in itself is a noteworthy phenomenon, an example of the confluence and conversation between science and dharma that is taking place in our time.^26^ Government and private funding for mindfulness research has followed a similar curve. The overall quality of the research, as in any field, could be described as “mixed.” However, as suggested above, it is increasingly becoming more rigorous, with more sophisticated study designs, active control conditions, investigation of underlying biological pathways through which mindfulness might be exerting its effects, and publication in top-tier journals.^27^

One of the original accelerants to this curve was the development of MBCT and the papers that have stemmed from its impact and investigation since the year 2000. Another accelerant comes from increasing interest in meditation among neuroscientists and from the studies they have pursued on various aspects of mindfulness and its possible neural correlates. This includes studies of structural and activation changes in the brain, investigation of possible mechanisms of action—including attention regulation, emotion regulation, perspective taking, social interconnectedness, and interpersonal neurobiology—as well as changes in functional connectivity between brain regions and networks. Other contributing accelerants to this curve at various times have included entire scientific journals as well as special issues of journals devoted to mindfulness research, major funding by NIH and other agencies, national and international conferences on the subject, and Mind and Life Institute-sponsored dialogs between scientists and philosophers and scholars, on the one hand, and the Dalai Lama and other contemplatives on the other.^28^

There is no question in my mind that the exponential increase in papers in the scientific and medical literature in the past 10 years, as shown in Fig. 1, is functioning, both for better and for worse, as a primary driver of worldwide interest in mindfulness. That being the case, it was virtually inevitable that, as noted earlier, “mindfulness” would sooner or later come to be hyped, commercialized, even exploited by some, especially by those who may not be grounded in ongoing personal practice but are just looking to capitalize on something that is trending hot at the moment. At the same time, it has generated a great deal of legitimate interest and coverage in the press and on television, some of it accurate and some of it not.

It was equally inevitable that the so-called “mindfulness revolution”^29^ would be criticized by others in a reactive backlash, perhaps without understanding the full dimensionality of the phenomenon in question, including the role of MBSR and its formulation from the beginning as a potentially transformative dharma vehicle, not only for individuals but for institutions and the larger society, with radical (in the sense of going to the root) implications for both social and political justice.^30^

These second order phenomena may be a transient social cost of “success.” I do think that the outsized popularity and hype accorded to mindfulness at the moment will be a passing fad, and that those with more opportunistic motivations will soon become bored and move on to the next next thing. Hopefully, “mindfulness” will fast become so “yesterday” to those interests. That will leave those of us who care deeply about it and see its potential for healing and liberation from suffering and the causes of suffering in one form or another to continue doing our work and cultivating our practice in ways that the world is actually starving for, and we might also say, literally and metaphorically, dying for. These are the stakes, in my view. In this regard, perhaps the less we use or overuse the word “mindfulness” and the more we embody it in our being and our doing, the better.

I have been told by Buddhist scholars that the dharma has fallen into decline many many times over the centuries in many of the cultures of Asia, only to reinvigorate itself and society cyclically in key moments when the conditions are right. This is probably one of those “key moments,” if not the most critical key moment ever on the planet, auguring a potential renaissance in all senses of the word, if we can come to grips with our own endemic enmity, fear, and self-centeredness as a species, as nations, and as individuals. Never have we needed the wisdom and freedom of the dharma and our inborn potential to realize it as we do now, for the sake of all beings and for the sake of the planet itself. So, taking certain risks to go beyond any parochial and fundamentalist perspectives we might harbor and deal directly with our own fears and our attachment to favored but necessarily limited views is what is called for in this era. And that includes our tendencies to fall into dogmatic, sectarian, or hopelessly dualistic perspectives—for instance, making “Buddhists” and “non-Buddhists,” or for that matter “us” and “them,” “the good guys and the bad guys”—in our own minds and then being attached to those distinctions in an absolutist way. This is the opposite of wisdom.

There are many different issues being debated nowadays in the scholarly community, such as whether MBPs have an ethical basis and whether they are optimal in terms of their explicit dharma content, or for that matter, in the “dosage” and duration of the meditation practices and the formats in which they are delivered. I certainly welcome empirical approaches to all these issues. Yet, misunderstandings and unwarranted assumptions abound. One example: the mischaracterization of contemporary mindfulness programs such as MBSR and MBCT as emphasizing *bare attention* (*sati*) without recognizing that they also include its complement, what is known as *clear comprehension* (*sampajañña*), in other words, discernment functioning together with attending (*satisampajañña*).^31^ This view may merely reflect a lack of familiarity with MBSR and its pedagogy. The same may be the case for those who assert that the Four Noble Truths and the Four Foundations of Mindfulness do not serve as the foundation of MBSR. In fact, they are the bedrock of MBSR, even though they are never mentioned. It is an empirical question as to whether they need to be explicit in an 8-week clinical or educational program designed to teach people how to take better care of themselves over the lifespan by developing a meditation practice. Certainly, some people who first encountered mindfulness through an MBP have sought out explicitly Buddhist teachings and communities and found a great value in doing so. Yet, it may be a conceit to imagine that making the dharma more explicit in a “second generation MBP” would necessarily add anything to the introduction of mindfulness within the mainstream for those with no interest in Buddhism or Dharma but who are suffering intensely from the slings and arrows, the full catastrophe, in Zorba’s term, of the human condition. Or it may not be. Certainly, there is room for an infinite number of imaginative approaches to healing the human condition.

## Conclusion

There has never been a better or more necessary time for all of us as human beings to wake up to our own collusion in the status quo, to the deep roots of self-centeredness and of subtle or not-so-subtle greed, hatred, and delusion within ourselves and our institutions, and to do what we can, being who we are, individually and collectively, for the sake of future generations as well as for our own—and even for ourselves as embodied individuals. Luckily, there is no essential separation between these. We only get this one moment, and this ever-so-brief human life to embody and live what we know and who we are, including the knowing of what we do not know—that is, to live our dharma as a radical act of sanity and love.

It seems to me that each one of us has a unique opportunity and a unique role to play in this unfolding, based on our love, our practice, and our unique karmic trajectory, grounded in our essential interconnectedness, non-separation, and common humanity. At this moment on the planet, we need all the various and disparate voices participating in this conversation, and we need to listen to each other with open hearts and deep attending. If we cannot do that, how could we possibly expect reconciliation across the greater divides of political and social animosity and active harming we are seeing enacted throughout the world today? This is the challenge and the promise of a democracy 2.0. This is the challenge of mindfulness and heartfulness embodied. It is up to us.

The dharma of course, whether with a big “D” or a little “d,” will take care of itself, as it always has. All we need to do is take care of what truly needs tending, with tenderness, and with resolve. And that is only everything.

How will it all unfold? You already know what is coming. Too early to tell.

## Endnotes

^1^ Apparently he mistakenly thought he was being asked about the 1968 street demonstrations in Paris.

^2^ Zhou Enlai apparently also acted decisively during the Cultural Revolution to save the magnificent 900-year-old Chan Buddhist Xiyan Temple in Suzhou by ordering it encircled by a brigade of the People’s Liberation Army. I was told this by Chan monks at the monastery who have participated in MBSR professional training programs.

^3^The same might be said of the American Revolution and our 250 year experiment with democracy, especially given the malaise and deep divides in views and perspectives underlying the 2016 US presidential election, to say nothing of the deep scars and enduring legacies of the Native American genocide that have never been fully owned or healed, the hundreds of years of the enslavement and trading of African human beings upon which the economy of the country was built and its legacies, and other similar historical patterns of othering, disregard, dehumanization, oppression, and exploitation ---See Zinn (2015).

^4^ The legendary and most likely mythological figure, said to have brought the dharma from India to China in the 5th Century, sat in a cave for nine years facing a wall, and eventually became the first Chan Patriarch in the Chan Mahayana tradition.

^5^ See p. 146 in Kabat-Zinn (2003).

^6^ See Kabat-Zinn (2013).

^7^ See p. 145 in Kabat-Zinn (2003). See also Kabat-Zinn (2005a).

^8^ See Condon et al. (2013).

^9^ See Kabat-Zinn (2005b). See also Dunne (2013).

^10^ See Joseph Goldstein (2002). See also Tanahashi (2014).

^11^ See Varela et al.(2017).

^12^ See, for example, Luk (1974).

^13^ This is clear in the ways that Bhikkhu Analayo, for instance, points out how all four satipatthanas partake of the same essence: “Each of them leads to liberation, like different gateways leading to the same city.” Anālayo (2003). See also p. 22, first paragraph. It is equally the case in the Anapansati Sutta (p. 21). Thich Nhat Hanh makes the same point regarding the Eightfold Noble Path factors; see Hanh (1998).

^14^ Respectfully “recontextualized” would be a more accurate way of framing it.

^15^ Had I decided, for purity’s sake, to call it “The Right-Mindfulness-Based Stress Reduction Program,” how much traction do you imagine it would have had in medicine in 1979? Or even now? Some things are better left implicit—and embodied. They are felt in the authentic presence of the teacher, and explicitly articulated and transmitted only as appropriate to the context of the moment. In MBP’s, many different domains of Buddhadharma can be implicitly present and embodied to whatever degree possible in the instructor, grounded in his or her own ongoing practice, and presence.

^16^ See http://www.umassmed.edu/cfm/stress-reduction/find-an-mbsr-program/ for MBSR instructors world wide certified by the University of Massachusetts Medical School’s Center for Mindfulness in Medicine, Health Care, and Society.

^17^ That is, to make meditation practice as American as anything else in our society that might have value as a lifestyle choice, such as regular exercise.

^18^ See Crane et al. (2016).

^19^ It was one way to introduce relatively rigorous formal meditative practices broadly in the society in the hope that they would take root and be incorporated into the unfolding daily lives of virtually everybody who is touched by their liberative potential. Thirty-eight years downstream from that effort, it has also led to introducing such practices in childhood through school-based programs.

^20^ For a compelling treatment of the value of mindfulness in medical practice across a wide array of disciplines and more generally, see Epstein (2017).

^21^ See for example: Crane et al. (2012), (2013).

^22^ In the sense of “extremely important and deserving of respect --- usually but not necessarily associated with religion”; “Veneration: A feeling of profound respect or reverence.” American Heritage Dictionary.

^23^ http://themindfulnessinitiative.org.uk/images/reports/Mindfulness-APPG-Report_Mindful-Nation-UK_Oct2015.pdf`

^24^ http://www.themindfulnessinitiative.org.uk/about/who-we-are

^25^ According to David Black of UCLA and MRM, who ran the data analysis, this graph is a trend estimate based on a title search of papers in the medical and scientific literature. It is a conservative estimate since mindfulness studies don't always use mindfulness in the title of the paper. It should be regarded as a snapshot in time (considering database updates, in press issue assignments, and other parameters). It will not map perfectly onto any other search at one time point, as all parameters are constantly updating. For this reason, year-to-year frequencies may differ when the citation numbers are recalculated. All in all, each curve of this kind is a trend estimate (David Black, personal communication Feb 27, 2017).

^26^ See for example: www.mindandlife.org; See also, W. Hasenkamp (Ed.), *The monastery and the microscope: Conversations with the Dalai Lama on mind, mindfulness, and the nature of reality*. New Haven, CT: Yale University Press.

^27^ See for example, Creswell (2017). Also see Goleman and Davidson (2017).

^28^ See Kabat-Zinn and Davidson (2011). Also see www.mindandlife.org and the collection of Mind and Life Institute volumes on dialogues with the Dalai Lama.

^29^ See Boyce (2011).

^30^ See for example, Kabat-Zinn (2011). See also: http://greatergood.berkeley.edu/article/item/how_mindfulness_can_defeat_racial_bias

^31^ See for example, Thera (2014).
